# Cartilage Homeostasis and Osteoarthritis

**DOI:** 10.3390/ijms23116316

**Published:** 2022-06-05

**Authors:** Yuta Fujii, Lin Liu, Lisa Yagasaki, Maiko Inotsume, Tomoki Chiba, Hiroshi Asahara

**Affiliations:** 1 Department of Systems Biomedicine, Tokyo Medical and Dental University, Bunkyo-ku, Tokyo 113-8501, Japan; y-fujii.syst@tmd.ac.jp (Y.F.); liulin.syst@tmd.ac.jp (L.L.); yagaperi@tmd.ac.jp (L.Y.); inotsume-m.syst@tmd.ac.jp (M.I.); chiba.syst@tmd.ac.jp (T.C.); 2 Department of Periodontology, Tokyo Medical and Dental University, Bunkyo-ku, Tokyo 113-851, Japan; 3 Department of Molecular and Experimental Medicine, The Scripps Research Institute, La Jolla, CA 92037, USA

**Keywords:** cartilage, osteoarthritis, Sox9, noncoding RNA, miRNA

## Abstract

Healthy limb joints are important for maintaining health and attaining longevity. Endochondral ossification (the replacement of cartilage with bone, occurring during skeletal development) is essential for bone formation, especially in long-axis bones. In contrast to endochondral ossification, chondrocyte populations in articular cartilage persist and maintain joint tissue into adulthood. Articular cartilage, a connective tissue consisting of chondrocytes and their surrounding extracellular matrices, plays an essential role in the mechanical cushioning of joints in postnatal locomotion. Osteoarthritis (OA) pathology relates to disruptions in the balance between anabolic and catabolic signals, that is, the loss of chondrocyte homeostasis due to aging or overuse of cartilages. The onset of OA increases with age, shortening a person’s healthy life expectancy. Although many people with OA experience pain, the mainstay of treatment is symptomatic therapy, and no fundamental treatment has yet been established. To establish regenerative or preventative therapies for cartilage diseases, further understanding of the mechanisms of cartilage development, morphosis, and homeostasis is required. In this review, we describe the general development of cartilage and OA pathology, followed by a discussion on anabolic and catabolic signals in cartilage homeostasis, mainly microRNAs.

## 1. Introduction

Osteoarthritis (OA) is the most common form of arthritis. It is characterized by gradual loss of articular cartilage, synovial membrane inflammation, osteophyte formation, and subchondral bone sclerosis. OA is associated with age-related loss of homeostatic balance between degradation and repair mechanisms in the articular cartilage [1,2,3,4,5]. This dysregulation induces senescence, differentiation, proliferation, and death in joint cells through gene and/or protein expression networks that switch from anabolic to catabolic outcomes. Cartilage-degrading enzymes, such as disintegrin and metalloproteinases with thrombospondin motifs (ADAMTS)-4 and ADAMTS-5, and matrix metalloproteinase (MMP)-13, are critical enzymes in OA pathogenesis [1,2,3]. Cartilage is composed of chondrocytes and an extracellular matrix whose major components include type II collagens and proteoglycans such as aggrecan. Chondrocytes in articular cartilage regulate cartilage homeostasis partly by synthesizing an extracellular matrix (ECM) rich in type II collagen, proteoglycans, and related macromolecules [6]. The advent of novel high throughput technologies has opened new perspectives in osteoarthritis research by mass spectrometry-based proteomic approaches [7]. In recent years, global proteomics studies using mass spectrometry have been widely conducted to elucidate the pathogenesis of articular cartilage [8]. Most studies have focused on proteins identified directly in the secretome of cartilage cell cultures [9,10,11,12,13,14,15,16,17,18,19], but proteomic analyses using cartilage tissue, cartilage explants [8], OA synovial fluid [20,21,22,23,24,25,26], and synovial cells have also been performed [8,27].

Once adult articular cartilage is damaged, its regeneration and repair are limited because of its hypovascularity. The catabolic and abnormal differentiation of chondrocytes due to aging or overuse of cartilage leads to loss of cartilage ECM, causing OA [28,29,30,31]. Articular cartilage maintains homeostasis by responding at the molecular level to various physiological stresses, including mechanical stress. When this balance is disrupted, osteoarthritis develops and progresses [32]. Aging is a major risk factor for OA, and aged chondrocytes exhibit multiple senescent phenotypes [33,34,35]. In particular, aging chondrocytes have reduced resistance to oxidative stress and impaired cellular homeostasis owing to autophagy dysfunction [36]. Synovitis, a common pathological change in osteoarthritic joints, is also a risk factor for OA, and severe synovitis exacerbates cartilage erosion [37,38]. Proinflammatory factors, such as interleukin-6 and tumor necrosis factor, are released from damaged joint tissue and induce synovial proliferation and inflammation, both known to contribute to synovitis [39]. OA can affect any joint but mainly affects the knee, hand, hip, and spine. The progressive and irreversible destruction of the cartilage matrix can cause joint pain and disability, which affects the quality of life [40,41]. Although many people with OA suffer from pain, available treatments in the early stage are limited to exercise therapy and symptomatic therapy, such as pharmacologic therapy, and joint replacement surgery is often indicated in the late phase of the disease. Fundamental treatments, such as pharmacologic interventions that could alter the progressive loss of articular cartilage and regenerate articular chondrocytes, are not available. To establish regenerative or preventative therapies for cartilage diseases, further understanding of the mechanisms of cartilage development, morphosis, and homeostasis is required. In this review article, we describe the general development of cartilage and OA pathology, followed by a discussion of anabolic and catabolic signals involved in cartilage homeostasis, focusing on noncoding RNAs (ncRNAs), especially microRNAs (miRNAs).

## 2. The Role of Sox9 on Chondrogenesis

SRY-Box Transcription Factor 9 (Sox9) is a member of the Sox family of transcription factors that are characterized by a high-mobility group (HMG)-box DNA-binding domain. Sox9 plays a pivotal role in male sex development because its sequence is at least 50% identical to that of the sex-determining factor SRY [42]. *Sox9* is expressed early in mesenchymal condensations throughout the embryo and is an essential cartilage-promoting factor during cartilage development and skeletal formation. To identify the role of *Sox9* in cartilage development, Bi et al. generated *Sox^−/−^* embryonic stem (ES) cells. The teratomas derived from *Sox9^−/−^* ES cells did not form any cartilage or expressed the chondrocyte-specific markers collagen type II alpha 1 (*Col2a1)*, collagen type IX alpha 2 (*Col9a2)*, collagen type XI alpha 2 (*Col11a2)*, or *Aggrecan* (*Acan*). In mouse chimeras, *Sox9^−/−^* cells were unable to express any chondrocyte-specific ECM genes, such as *Col2a1* [43]. In subsequent studies, *Sox9* heterozygous (*Sox9^+/−^*) mice showed cartilage-related defects and died shortly after birth [44]. These studies demonstrate that Sox9 plays a key role in chondrocyte differentiation and cartilage formation.

The chondrocyte differentiation marker *Col2a1*, which is expressed abundantly in the early stages of embryo development, colocalizes with *Sox9* in all chondroprogenitor cells. *Col2a1* encodes type II collagen, which is a major structural component of the cartilage. It is expressed in chondrogenic tissues before chondrocyte differentiation [45]. Sox9 binds directly to the *Col2a1* enhancer element to guide the transcription of the gene in chondrocytes. When chondrocytes hypertrophy in the growth plate, collagen type X alpha 1 (*Col10a1)* expression is activated. The Sox trio then disappears simultaneously, and *Col2a1* slowly disappears. *Col1a1* expression is activated as cartilage is replaced by bone [46]. When Sox9 activity is reduced, the production of cartilage matrix proteins such as type II collagen is inhibited, leading to major skeletal abnormalities [47].

Several studies show that SRY-Box Transcription Factor 5 (Sox5) and SRY-Box Transcription Factor 6 (Sox6) can activate Sox9 in developing cartilage cells [48,49,50]. Researchers also found that L-Sox5 (a new form of Sox5) and Sox6 are coexpressed with Sox9 during chondrogenesis and that these three Sox transcription factors cooperate with each other in the activation of the chondrocyte differentiation marker *Col2a1* [46,51,52].

## 3. The Effect of Sox9 on Cartilage Homeostasis and OA

Cartilage is vital throughout vertebrate life, and Sox9 is essential for cartilage development. In addition, Acan is a major ECM protein of both the growth plate and articular cartilage, and its expression has been detected in all articular cartilages throughout development and beyond [53]. To examine the function of Sox9 postnatally, Haseeb et al. generated a cartilage-specific *Sox9* conditional knockout *Sox9^fl/fl^;Acan^CreERT2/+^* mice line, which, when given a tamoxifen shot, deletes *Sox9*. In 3-month-old *Sox9*-deleted mutant mice, the loss of proteoglycans and hypertrophic zones was observed. Additionally, upon performing destabilization of the medial meniscus (DMM) surgery on experimental mice, the Osteoarthritis Research Society International (OARSI) scale was found to be higher in *Sox9*-deleted mutant mice than in control mice. In conclusion, Sox9 is required to keep growth plates open and articular cartilage resistant to OA [54,55]. These results confirmed that Sox9 plays a vital role postnatally.

Oh et al. performed chromatin immunoprecipitation sequencing (ChIP-Seq) to identify genes that harbor Sox9-interaction sites and RNA sequencing to identify genes affected by Sox9. Their results show that Sox9 regulates a specific set of cartilage ECM genes, including *Acan* and *Col2a1*, and controls the differentiation of cartilage ECM cells [21,56]. Additionally, a recent ChIP-seq analysis using the CRISPR/Cas system revealed the existence of a rib cage-specific enhancer (RCSE) located approximately 1 Mb upstream of *Sox9* [57]. Multiple additional analyses with CRISPR-ChIP-mass spectrometry (CRISPR-ChIP-MS) demonstrated that the transcription factor STAT3 regulates the expression of *Sox9* via this RCSE region [57]. As chondrocytes are the only cell type in the cartilage ECM, cartilage repair is highly dependent on correct chondrogenic differentiation of resident progenitor cells and ECM anabolism by differentiated chondrocytes [22,58]. Studies have demonstrated that upregulation of *SOX9* could inhibit IL-1β-induced inflammation in human chondrocytes, and *SOX9* transduction can renew the capacity of late passage human OA articular chondrocytes to form cartilage ECM [59]. Tankyrase-mediated poly(ADP-ribosyl)ation (PARylation) of Sox9 plays an essential role in the regulation of Sox9 ubiquitination and degradation. Sox9 binds to *Tnks* and *Tnks2,* which encode tankyrase, a regulator of cartilage matrix anabolism. Inhibition of tankyrase increases *Sox9* expression, promotes cartilage ECM synthesis, and enhances chondrogenic differentiation of mesenchymal stem cells. Delivery of tankyrase inhibitors can prevent OA in mouse knee joints [60]. Thus, these findings could be applied to future studies on OA therapeutic potential.

## 4. ncRNAs Involved in Cartilage Homeostasis and OA

More than 90% of the human DNA is actively transcribed, however, only 2% encodes proteins. The majority of the transcripts are ncRNAs. They are classified according to their biosynthesis, length, and mechanisms of action. After transcription, ncRNAs may form short, long, and circular ncRNAs with unique secondary and tertiary structures. ncRNAs are transcribed but not translated into proteins and perform their biological functions at the RNA level [61]. Short ncRNAs are less than 200 nucleotides in length, and include miRNAs, small nucleolar RNAs (snoRNAs), Piwi-interacting RNAs (piRNAs), small interfering RNAs (siRNAs), transition RNAs (tRNAs), tRNA-derived fragments (tRFs), and Y RNA fragments. Among short ncRNAs, miRNAs are the most frequently studied, but there have also been reports on other short ncRNAs associated with OA.

After the first profiling study on snoRNAs altered in OA was performed, several snoRNAs were studied through loss-of-function and gain-of-function experiments [62]. Differentially expressed snoRNAs have been identified in aging and OA, and their knockdown or overexpression have been shown to alter the expression of chondrogenesis, cartilage hyperplasia, and OA-related genes [62].

tRFs are novel regulators of post-transcriptional gene expression. However, their expression profiles and role in post-transcriptional gene regulation in chondrocytes are unknown. In 2020, a tRF in the cartilage was reported for the first time. The expression profile of tRFs is altered in OA cartilage and chondrocytes stimulated with IL-1b, whereas tRF-3003a represses Janus kinase 3 (*JAK3*) gene expression in chondrocytes [63]. In addition, the expression of specific tRFs was shown to be different in old chondrocytes compared to young chondrocytes [64,65].

Recently, there has been a growing interest in competing endogenous RNAs (ceRNAs), such as circular RNAs (circRNAs) and long ncRNAs (lncRNAs), which act as miRNA sponges, although the physiological significance of ceRNAs is not well understood. Several ncRNAs have been reported to be involved in cartilage development and homeostasis [66].

### 4.1. MiRNAs

MiRNAs are short ncRNAs that regulate gene expression by altering target mRNA stability and inhibiting protein synthesis. In most cases, miRNAs are transcribed into primary miRNAs (pri-miRNAs) by RNA polymerase II. Pri-miRNAs have a cap structure, a poly-A tail, and a loop structure. Both the cap structure and poly-A tail are cleaved by the ribonuclease enzyme III Drosha to form a precursor miRNA (pre-miRNA) that is transported to the cytoplasm by exportin 5. When the loop structure is cleaved by Dicer, a ribonuclease enzyme III, mature miRNA is generated.

MiRNAs have been found to be important for mammalian development. In 2005, Dicer was found to be essential for vertebrate limb morphogenesis. Targeted removal of Dicer in mouse limbs results in the formation of much smaller limbs [67]. The importance of Dicer in skeletal development was confirmed in 2008 when mice with their *Dicer* gene deleted in cartilage showed a reduction in the proliferation of chondrocytes in *Dicer*-null growth plates. Severe skeletal developmental defects were also observed [68]. Iliopoulos et al. screened 365 miRNA genes and found 16 miRNA gene signatures that were differentially expressed in OA [69]. Using bioinformatics analysis, Cong et al. identified 46 differentially expressed miRNAs involved in chondrocyte apoptosis, autophagy, differentiation, and ECM degradation [70].

#### 4.1.1. MiRNA-140

Several miRNAs play important roles in cartilage development and homeostasis. MiRNA-140 (miR-140) is known to be a cartilage-specific miRNA in embryos and zebrafish and a critical regulator of cartilage development and homeostasis [71,72,73,74]. It was also found that miR-140 expression was reduced in human OA cartilage [69,73]. MiR-140 is located in intron 16 of the WW domain-containing protein 2 (*Wwp2*), which is a member of the C2-WW-HECT family (NEDD4 family) of E3 ubiquitin ligases [75]. It acts as an acceptor of ubiquitin from E2 enzymes and then transfers ubiquitin to a specific lysine residue on the substrate [76], which is an intronic miRNA [50]. The expression level of *Wwp2*, the host gene of miR-140, is reduced in OA.

**Figure 1:**
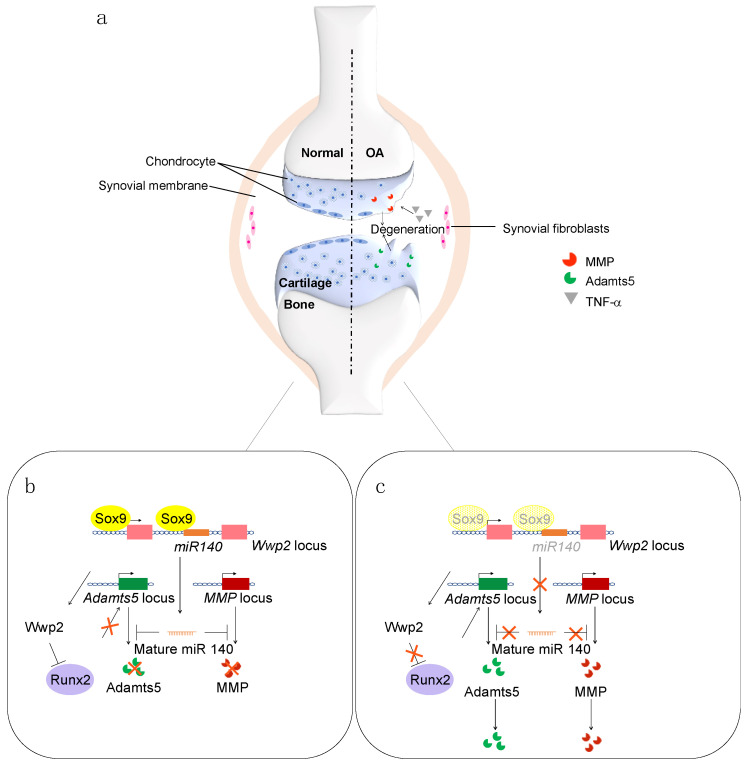
(**a**) Schematic representation of normal (left) and OA (right) cartilages. (**b**) Cartilage homeostasis is maintained by the Sox9 and miR-140 pathways. (**c**) In OA, the expression levels of Sox9 and miR-140 are decreased.

The deletion of Sox9 diminishes the expression of miR-140 [74]. The miR-140 primary transcript is an intron-retained RNA coexpressed with *Wwp2*, which is directly induced by Sox9 through binding to intron 10 of the *Wwp2* gene during chondrogenesis [77]. Nakamura et al. identified several Sox9 binding sites upstream of that for miR-140 within the *Wwp2* gene in humans, mice, and zebrafish, and showed that miR-140 is downstream of the transcription factor Sox9 in developing zebrafish and mammalian cells [78]. Yamashita et al. demonstrated that the proximal upstream region of pri-miR-140, located in intron 10 of *Wwp2*, has in vivo promoter activity. These results suggest that miR-140 may be derived from its own specific transcript via Sox9 binding during chondrogenesis (Figure 1) [50]. Sox5 and Sox6 also function as regulators of cartilage development by boosting Sox9 activation of *Col2a1* and *Agc1* [46,79]. Yamashita et al. showed that the DNA-binding and/or transactivation ability of Sox9 as a homodimer is boosted by Sox5 and Sox6 in the promoter region of pri-miR-140 [50].

To further understand the function of chondrocyte-specific miR-140, Miyaki et al. and Nakamura et al. generated miR-140 deletions in mice. These mice exhibited craniofacial truncation due to impaired chondrocyte differentiation [74,80]. Interestingly, *Wwp2* knockout (KO) mice have been reported to exhibit a similar craniofacial truncation phenotype [81]. These reports suggest that miR-140 and its host gene *Wwp2* have overlapping or coherent functions in craniofacial morphogenesis. However, this phenotype might be due to the loss of miR-140 because a gene-trap technology was used in the corresponding studies to generate *Wwp2*-null mice, preventing the expression of exons and introns downstream of the insertion site of the gene-trap cassette. To explore this question, Inui et al. generated *Wwp2* and/or mir-140 KO mice using the CRISPR/Cas9 system. They confirmed that the skulls of miR-140 KO mice were truncated, as previously reported [74,80]. However, the skulls of *Wwp2* KO mice were indistinguishable from those of wild-type (WT) mice. These results suggest that miR-140 is required for proper craniofacial development but that the Wwp2 protein is not [82]. Although we did not find a cooperative function between miR-140 and Wwp2 in craniofacial development in mice, it remains possible that these two factors cooperate in other contexts in mammals. Wwp2 protects articular cartilage by regulating Adamts5 via the Wwp2-Runx2 pathway. The regulation of a common target by both Wwp2 and miR-140 might cooperatively enhance their function in maintaining cartilage homeostasis at both the pre- and post-transcriptional stages [83].

#### 4.1.2. MiR-455

MiR-455, located in intron 10 of collagen type XXVII alpha 1 (*Col27a1*) [84] has been expressed in cell culture models of chondrogenesis (along with miR-140). It regulates transforming growth factor (TGF)-β signaling by suppressing the Smad2/3 pathway [85]. MiR-455-3p has an important role in the regulation of chondrogenic differentiation of human adipose-derived stem cells (hADSCs) [86]. In addition, miR-455-3p regulates OA and the chondrogenic differentiation of human mesenchymal stem cells (hMSCs) [87,88]. Moreover, miR-455-3p functions as an activator of early chondrogenic differentiation by promoting the expression of the cartilage-specific genes *Col2a1* and *Comp* and directly targeting and inhibiting Runt-related transcription factor 2 (*Runx2*) [87]. MiR-455-3p promotes chondrogenic differentiation by suppressing the expression of histone deacetylase (*HDAC)2* and *HDAC8*, thereby maintaining an appropriate level of histone H3 acetylation at the *COL2A1* promoter to promote the production of type II collagen [88]. Furthermore, it has been shown that miR-455-3p can regulate hMSC chondrogenic differentiation by directly targeting the DNA methyltransferase *(DNMT) 3A* 3′-UTR [88]. Studies have also shown that miR-455-3p promotes TGF-β signaling and inhibits cartilage degeneration by directly targeting P21-activated kinase (*PAK2*) [89]. In miR-455-3p global KO mice, obtained by using a transcription activator-like effector nuclease system, thinner cartilage thickness was observed compared to that in WT mice at six months of age [90]. Moreover, these mice showed an OA-like phenotype at five months of age, indicating that *miR-455-3p* is a critical regulator of cartilage homeostasis [89].

Intronic miRNAs are believed to be processed from the introns of their host transcription units and hence share common regulatory mechanisms and expression patterns with the host gene [91,92,93]. We have previously reported that miR-140 may be derived from its own specific transcript via Sox9 binding during chondrogenesis [50]. To investigate other miRNAs regulated by Sox9 in chondrocytes, a comprehensive microarray analysis was performed. Among several candidates, miRNA-455 showed enhanced expression from both 5p and 3p strands in a Sox9 concentration-dependent manner [94] and was expressed in chondrocytes in approximately equal amounts from both strands [94]. To investigate whether this was directly regulated by Sox9, we used ChIp analysis and found a binding site for Sox9 within intron 3 of *Col27a1* [94]. Usually, only one strand of miRNA is incorporated into the RNA-induced silencing complex (RISC) to form a functional and mature miRNA complex [95,96,97]. However, recent reports noted that, in exceptional cases, two distinct miRNAs can be generated, although their functional relevance is not fully understood [98,99,100,101]. In order to investigate the in vivo function of miR-455, we generated miR-455 KO mice using the CRISPR/Cas9 method. Although miR-455^−/^^−^ mice were born with a normal appearance and showed a normal skeletal development, their knee joints showed cartilage disruption at six months of age. This was consistent with observations from a previous study [89].

We then screened for miR-455 targets using a reporter library system and identified several previously unreported miR-455 target candidates. We focused on endothelial PAS domain protein 1 (*EPAS1)*, which encodes hypoxia inducible factor (HIF)-2α and has a seed sequence in the 3′-UTR for both miR-455s. HIF-2α is known as a catabolic transcription factor for cartilage homeostasis [102,103]. We revealed that both miR-455-5p and -3p directly regulate *EPAS1* expression, suggesting that both miR-455s have anti-inflammatory functions and protect against cartilage destruction in OA. To investigate the potential therapeutic effects of miR-455s, we used the well-established surgical DMM model of OA injected with miR-455s mimics. Injection of both miR-455-5p and -3p mimics into DMM-treated knee joints significantly inhibited cartilage destruction compared to the results from the injection of control mimics. These results reveal a therapeutic effect of miR-455-5p and -3p in treating cartilage degeneration in OA, possibly by repressing Hif-2α expression [94]. Hif-2α is a potential therapeutic target for OA since it is encoded by *EPAS1*, an important developmental gene. *Epas1*^−/^^−^ mice are embryonically lethal, whereas *Epas1*^+/^^−^ mice show dwarfism [103]. Therefore, indirect suppression of *EPAS1* by miR-455s may be a safe treatment for OA.

#### 4.1.3. MiRNAs Regulating Hif-2α

Other miRNAs regulate Hif-2α expression. Zhou et al. revealed that the inhibition of *SDC-4* affects cartilage homeostasis and improves the chondrocyte hypertrophy phenotype by inducing miR-96-5p expression. miR-95-5p targets *HIF-2α* 3′-UTR sequences and thus inhibits Hif-2α translation in murine cartilage tissue and chondrocytes [104]. MiR-365 downregulates *HDAC4* and decreases chondrocyte hypertrophy; therefore, miR-365 is an important regulator of chondrocyte hypertrophy and differentiation [105]. Hwang et al. demonstrated that miR-365 levels were significantly suppressed in OA cartilage and that IL-1β decreased miR-365 levels in articular chondrocytes through the activation of the MAPK and NF-κB signaling pathways. MiR-365 suppresses IL-1β-mediated catabolic responses in monolayer and 3D cultures of articular chondrocytes, with concurrent regulation of HIF-2α expression, suggesting that miR-365 could be a useful target for OA therapy [106].

The Sp-1 and Hif-2α protein concentration reduction that occurs after overexpressing miR-138 leads to a marked reduction in the expression of major matrix collagen, COL2A1, which is critical for normal cartilage structure and function [107].

#### 4.1.4. Other miRNAs

Several other miRNAs have been associated with growth plate maintenance and OA development. MiRNA-322 is strongly expressed in prehypertrophic to hypertrophic zones [108] and regulates the RAF/MEK/ERK pathway [109]. A disruption in this pathway in cartilage tissues causes cartilage dysplasia [110]. An analysis of cartilage tissues from miRNA-322-deficient mice (generated with the CRE-loxP system) revealed that hemizygous mutants died neonatally due to respiratory failure resulting from tracheal cartilage damage [108]. In the growth plate, miRNA-322-deficient mice exhibit a slightly reduced hypertrophic zone phenotype [108].

Growth-arrest-specific 5 (GAS5), an lncRNA, plays an important role in mammalian growth and differentiation [111]. GAS5 acts as a negative regulator of miR-21 and causes OA [112]. In chondrosarcoma-derived chondrocytes (HCS-2/8 cells) overexpressing GAS5, miR-21 expression is downregulated. Conversely, knockdown of miR-21 upregulates GAS5 [113]. In a rat model overexpressing GAS5, miR-21 expression is suppressed in growth plate chondrocytes. As a result, cell proliferation is suppressed, and apoptosis is promoted [113], suggesting that miR-21 may play an important role in the maintenance and differentiation of chondrocytes in the growth plate.

MiR-17 belongs to the miR-17-92 cluster [114]. The dysregulation of this miRNA cluster has been associated with skeletal malformations and related growth defects in humans [115]. However, the function of the miR-17-92 cluster, especially that of miR-17, in adult cartilage maintenance and OA progression has not been fully elucidated. Recently, it was reported that decreased expression of miR-17, which targets pathological catabolic factors, including MMP-3, MMP-13, ADAMTS5, and NOS2, in osteoarthritic chondrocytes, contributes to OA progression. Furthermore, miR-17 is highly expressed in both superficial and middle chondrocytes under physiological conditions and maintains the physiological catabolic and anabolic balance, potentially by restricting HIF-1α signaling. Therefore, miR-17 has dual functions: It maintains cartilage homeostasis and prevents OA [116].

Mir-379-5p, located on chromosome 14q32.31 [117], is downregulated in human osteoarthritic tissue and negatively correlated with *YBX1* expression. Treating chondrocytes with IL-1β resulted in high expression of mir-379-5p, increased cell viability, increased levels of proliferation-related proteins, and overexpression of ECM-related proteins, such as collagen II and aggrecan. It also results in decreased expression of inflammatory factors and ECM-related proteins, such as MMP-1 and MMP-13. Luciferase reporter assays validated the relationship between miR-379-5p and *YBX1*. This function was demonstrated via the PI3K/Akt pathway and inhibited by a PI3K/Akt pathway inhibitor. These results indicate that miR-379-5p promotes the proliferation of articular chondrocytes in OA by interacting with *YBX1* and regulating the PI3K/Akt pathway [118].

### 4.2. CircRNAs

CircRNAs are generated by back splicing and discriminated by a covalently closed-loop structure without either a 5′-3′ polyadenylated or polar tail [119,120,121]. CircRNAs derive from known protein-coding genes that comprise one or more exons. Notably, they are exceptionally stable due to their loop structures [122]. In 1976, Sanger et al. identified the first circRNAs, viroids from RNA viruses, using an electron microscope [123]. For a long time, circRNAs were regarded as transcriptional noise produced during abnormal splicing. Advances in biological research using next-generation sequencing have identified thousands of new circRNAs functionally annotated in multiple physiological and pathological processes in eukaryotes, including cancer progression [124,125], inflammation [126], aging [127], and infection [128]. Associations between circRNAs and cartilage metabolism and OA have also been reported.

#### 4.2.1. CircRNAs and Idiopathic Short Stature (ISS)

In patients with ISS, 83 and 62 circRNAs were up and downregulated, respectively, compared with those in healthy controls. One of the circRNAs that was highly expressed in ISS, circRNA_0079201, functions as an miR-140-3p sponge. Furthermore, the proliferation, hypertrophy, and endochondral ossification of chondrocytes in ISS are regulated by the hsa_circRNA_0079201/miR-140-3p/SMAD2 pathway [129].

#### 4.2.2. CircRNAs and OA

Differences in circRNA expression in the healthy cartilage of patients with OA have been reported. Whole transcriptome sequencing revealed that the expression of 42 circRNAs was altered in OA cartilage tissues compared with that in normal cartilage tissues [130]. It was also observed that the expression of 1380 circRNAs differed between OA and control chondrocytes [131]. Subsequently, Xiao et al. identified 197 differentially expressed circRNAs in OA knee joints [132]. Furthermore, 119 upregulated and 136 downregulated circRNAs were identified by RNA-seq in an OA mouse model induced by IL-1β [133]. A total of 11 downregulated and 101 upregulated circRNAs were identified in OA cartilage [134]. These changes in circRNA expression patterns indicate a potential function in OA. The relationship between OA and circRNAs has been gradually elucidated through mechanisms such as circRNA interference with chondrocyte proliferation and apoptosis [135], regulation of ECM degradation [136], and inflammation [137]. In the last few years, an increasing number of associations between circRNAs and OA have been reported. Here, we briefly review the latest literature. For example, circSEC24A is upregulated in OA cartilage tissues and chondrocytes. This upregulation aggravates IL-1β-induced injury by downregulating IL-1β and reducing miR-142-5p in IL-1β-stimulated chondrocytes [138]. CircSCAPER promotes IL-1β-induced ECM degradation, proliferation arrest, and apoptosis enhancement in human chondrocytes by regulating the miR-140-3p/EZH2 axis [139]. The circRNA derived from vacuolar ATPase assembly factor (*VMA21*) suppresses LPS-induced chondrocyte apoptosis in OA by decreasing the production of mature miR-103 [140]. Circ_0020014 acts as an miR-613 sponge to regulate *ADAMTS5* expression, thereby protecting chondrocytes from IL-1β-induced inflammatory damage [141]. In addition, circ-LRP1B regulates proliferation, apoptosis, and oxidative stress in LPS-stimulated human C28/I2 chondrocytes via the miR-34a-5p/NRF1 network [142]. CircRHOT1 enhances CCND1 expression by sponging miR-142-5p to inhibit chondrocyte autophagy and promote chondrocyte proliferation in OA [143]. Circ_0005526 promotes IL-1β-induced chondrocyte injury in OA by suppressing miR-142-5p binding to transcription factor 4 [144]. Circ_0043947 contributes to interleukin 1β-induced chondrocyte injury by sponging miR-671-5p to upregulate reticulon 3 expression [145]. CircADAMTS6/miR-324-5p/PIK3R3 axis participate in IL-1β-induced human chondrocyte dysfunction via the PI3K/AKT/mTOR signaling pathway [146]. Besides, the circ_0000423/miR-27b-3p/MMP-13 axis can affect the pathogenesis of OA [134]. Nevertheless, further research on the relationship between circRNAs and OA is required.

### 4.3. MiRNAs and Diseases

Dysregulation of miRNAs is observed in a variety of diseases, including cancer. However, only a few congenital diseases associated with mutations in miRNA genes or their target regions have been reported.

### 4.3.1. Disease-Related miRNAs

The first report of a genetic disease involving miRNAs showed that point mutations in the seed region of miR-96, an miRNA expressed in the hair cells of the inner ear, resulting in autosomal dominant, progressive hearing loss [147].

#### 4.3.2. MiRNAs and Skeletal Dysplasia

The Nosology and Classification of Genetic Skeletal Disorders published by the Nosology Committee of the International Skeletal Dysplasia Society comprises 461 different diseases classified into 42 groups based on their clinical, radiographic, and/or molecular phenotypes. Remarkably, pathogenic variants affecting 437 different genes have been found in 425/461 (92%) of these disorders [148]. However, the underlying molecular mechanisms have not been elucidated for many of them. Noteworthy, miRNAs have been associated with skeletal dysplasia.

The miR-17-92 cluster gene Mir17HG was the first miRNA-encoding gene whose mutation was found to cause abnormal skeletal development in humans. Its deletion causes the arm-length syndrome (Feingold syndrome type 2) [115]. The skeletal phenotype varies from case to case, one family shows skeletal overgrowth with polydactyly [149], while another study reported a case of a nine-year-old boy with developmental delay, short stature, mild macrocephaly, hypertelorism, brachydactyly, and clinodactyly [150]. The reason for these phenotypic inconsistencies is unclear. It might be explained by differences in genetic and non-genetic factors specific to each case, including the miR-17-92 expression level.

#### 4.3.3. Gain-of-Function Mutation of miR-140

It is well known that a deficiency in miR-140 causes skeletal dysplasia. Recently, Gliogieniene et al. reported new findings on skeletal dysplasia caused by a point mutation in the miR-140 gene and on the underlying mechanism. They discovered that, in a novel autosomal dominant human skeletal dysplasia, a single nucleotide substitution occurs in miR-140, resulting in a neomorphic (gain-of-function) mutation. A single nucleotide substitution in miR-140-5p was identified by whole-genome sequencing of an ultra-rare congenital skeletal disorder [151]. Since miR-140 is completely conserved in vertebrates, a knock-in mouse model was generated with the same base substitution to examine its effect on mice. Mutant mice showed abnormalities similar to those in the patient’s skeleton, proving the causative role of this variant. Interestingly, the cartilage phenotype of miR-140^G/G^ mice differed from that of miR-140-null (miR140^−/^^−^) mice [74,80,151]. MiR-140 mutated mice displayed a decrease in *Col10a1* expression, a delay in secondary ossification of the carpal and tubular bones, a severe decline in epiphyseal mineralization, and a mild flat vertebral body. These phenotypes match the features of patients with skeletal dysplasia [151].

Furthermore, this mutant miRNA gene resulted in abundant mutant miR-140-5p expression without defects in miRNA processing. In chondrocytes, the mutation extensively derepressed the targets of WT miR-140-5p and represses those of mutant miR-140-5p, suggesting that the mutation has both loss-of-function and gain-of-function effects. Furthermore, mutant miR-140-5p competes with Ybx1, a conserved RNA-binding protein, for an overlapping binding site. This may explain why this mutant miRNA strongly represses its targets and exerts robust effects in vivo, even in the absence of evolutionarily selected miRNA-target RNA interactions [152,153]. This is the first reported case of a pathogenic gain-of-function miRNA mutation. It provides molecular insights into the novel actions of emerging or mutant miRNAs [151].

## 5. Conclusions

This review focuses on Sox9, the master transcription factor of cartilage, and ncRNAs, mainly miRNAs, and provides an overview of previous reports. To date, many factors involved in the maintenance of cartilage homeostasis and OA have been identified, but our understanding of the interactions and networks among these factors remains incomplete. It is hoped that the powerful tools that have emerged in recent years will lead to greater knowledge of the mechanisms of musculoskeletal congenital diseases and, ultimately, to therapeutic approaches.

## Data Availability

Not applicable.

## Abbreviations

*Acan*	*Aggrecan*
ADAMTS	a disintegrin and metalloproteinases with thrombospondin motif
ceRNA	competing endogenous RNA
ChIP-Seq	chromatin immunoprecipitation sequencing
circRNA	circular RNA
Col2a1	collagen type II alpha 1
Col9a2	collagen type IX alpha 2
Col11a2	collagen type XI alpha 2
Col10a1	collagen type X alpha 1
Col27a1	collagen type XXVII alpha 1
ceRNA	competing endogenous RNA
CRISPR-ChIP-MS	CRISPR-ChIP-mass spectrometry
DMM	destabilization of the medial meniscus
DNMT	DNA methyltransferase
ECM	extracellular matrix
EPAS1	endothelial PAS domain protein 1
ES	embryonic stem
GAS5	Growth-arrest-specific 5
hADSC	human adipose-derived stem cell
HDAC	histone deacetylase
HMG	high-mobility group
HIF	hypoxia inducible factor
hMSC	human mesenchymal stem cell
ISS	idiopathic short stature
JAK3	Janus kinase 3
KO	knockout
lncRNA	long ncRNA
miR-140	MiRNA-140
miRNA	microRNA
MMP	matrix metalloproteinase
ncRNAs	noncoding RNAs
OA	Osteoarthritis
OARSI	Osteoarthritis Research Society International
PAK2	P21-activated kinase
PARylation	poly(ADP-ribosyl)ation
piRNAs	Piwi-interacting RNAs
pre-miRNA	precursor miRNA
pri-miRNA	primary miRNA
RCSE	rib cage-specific enhancer
RISC	RNA-induced silencing complex
Runx2	Runt-related transcription factor 2
siRNA	small interfering RNA
snoRNA	small nucleolar RNA
Sox5	SRY-box transcription factor 5
Sox6	SRY-box transcription factor 6
Sox9	SRY-box transcription factor 9
tRF	tRNA-derived fragment
tRNA	transition RNA
TGFβ	transforming growth factor-β
VMA21	vacuolar ATPase assembly factor 21
WT	wild-type
Wwp2	WW domain-containing protein 2
